# Polygenic risk for schizophrenia, social dispositions, and pace of epigenetic aging: Results from the Young Finns Study

**DOI:** 10.1111/acel.14052

**Published:** 2023-11-29

**Authors:** Aino Saarinen, Saara Marttila, Pashupati P. Mishra, Leo‐Pekka Lyytikäinen, Emma Raitoharju, Nina Mononen, Elina Sormunen, Mika Kähönen, Olli Raitakari, Jarmo Hietala, Liisa Keltikangas‐Järvinen, Terho Lehtimäki

**Affiliations:** ^1^ Department of Psychology and Logopedics, Faculty of Medicine University of Helsinki Helsinki Finland; ^2^ Helsinki University Central Hospital Adolescent Psychiatry Outpatient Clinic Helsinki Finland; ^3^ Department of Molecular Epidemiology, Faculty of Medicine and Health Technology Tampere University Tampere Finland; ^4^ Gerontology Research Center Tampere University Tampere Finland; ^5^ Faculty of Medicine and Health Technology Tampere University Tampere Finland; ^6^ Department of Clinical Chemistry Fimlab Laboratories Tampere Finland; ^7^ Finnish Cardiovascular Research Center Tampere Finland; ^8^ Department of Cardiology, Heart Center Tampere University Hospital Tampere Finland; ^9^ Cardiovascular Research Center Tampere, Faculty of Medicine and Health Technology Tampere University Tampere Finland; ^10^ Department of Psychiatry University of Turku Turku Finland; ^11^ Turku University Hospital Turku Finland; ^12^ Department of Clinical Physiology Tampere University Hospital Tampere Finland; ^13^ Research Centre of Applied and Preventive Cardiovascular Medicine University of Turku Turku Finland; ^14^ Centre for Population Health Research University of Turku Turku Finland; ^15^ Department of Clinical Physiology and Nuclear Medicine Turku University Hospital Turku Finland; ^16^ Department of Medicine University of Turku Turku Finland; ^17^ Division of Medicine Turku University Hospital Turku Finland

**Keywords:** biological clock, epigenetic clock, longitudinal, psychosis, social development, social functioning

## Abstract

Schizophrenia is often regarded as a disorder of premature aging. We investigated (a) whether polygenic risk for schizophrenia (PRS_sch_) relates to pace of epigenetic aging and (b) whether personal dispositions toward active and emotionally close relationships protect against accelerated epigenetic aging in individuals with high PRS_sch_. The sample came from the population‐based Young Finns Study (*n* = 1348). Epigenetic aging was measured with DNA methylation aging algorithms such as AgeAccel_Hannum_, EEAA_Hannum_, IEAA_Hannum_, IEAA_Horvath_, AgeAccel_Horvath_, AgeAccel_Pheno_, AgeAccel_Grim_, and DunedinPACE. A PRS_sch_ was calculated using summary statistics from the most comprehensive genome‐wide association study of schizophrenia to date. Social dispositions were assessed in terms of extraversion, sociability, reward dependence, cooperativeness, and attachment security. We found that PRS_sch_ did not have a statistically significant effect on any studied indicator of epigenetic aging. Instead, PRS_sch_ had a significant interaction with reward dependence (*p* = 0.001–0.004), cooperation (*p* = 0.009–0.020), extraversion (*p* = 0.019–0.041), sociability (*p* = 0.003–0.016), and attachment security (*p* = 0.007–0.014) in predicting AgeAccel_Hannum_, EEAA_Hannum_, or IEAA_Hannum_. Specifically, participants with high PRS_sch_ appeared to display accelerated epigenetic aging at higher (vs. lower) levels of extraversion, sociability, attachment security, reward dependence, and cooperativeness. A rather opposite pattern was evident for those with low PRS_sch_. No such interactions were evident when predicting the other indicators of epigenetic aging. In conclusion, against our hypothesis, frequent social interactions may relate to accelerated epigenetic aging in individuals at risk for psychosis. We speculate that this may be explained by social‐cognitive impairments (perceiving social situations as overwhelming or excessively arousing) or ending up in less supportive or deviant social groups.

## Graphical abstract

Individuals with a high polygenic risk for schizophrenia seem to display accelerated epigenetic aging at higher (vs. lower) inner drive for social relationships. A rather opposite pattern was found among those with a low polygenic risk for schizophrenia. Thus, frequent social interactions may relate to accelerated epigenetic aging in individuals at risk for psychosis.

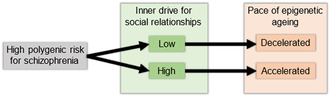

**Abbreviations**
DSM: Diagnostic and Statistical Manual of Mental Disorders
EAS: the Emotionality, Activity, and Sociability Temperament Survey
GWAS: genome‐wide association study
HWE: Hardy–Weinberg Equilibrium
ICD: International Statistical Classification of Diseases and Related Health Problems
MAF: minor allele frequency
MSPSS: the Multidimensional Scale of Perceived Social Support
NEO‐FFI: the Neuroticism‐Extraversion‐Openness Five‐Factor Inventory
PRS_DEP_
: polygenic risk for major depression
PRS_SCZ_
: polygenic risk for schizophrenia
SNP: single‐nucleotide polymorphism
TCI: The Temperament and Character Inventory
YFS: the Young Finns Study

## 1 INTRODUCTION

It is well known that schizophrenia patients display many signs of premature aging. For example, compared with the general population, schizophrenia patients are estimated to have 9–18 years shorter expected lifespan (Plana‐Ripoll et al., 2019), especially if they have comorbid psychiatric disorders (Plana‐Ripoll et al., 2020). Also, schizophrenia patients show accelerated aging in terms of metabolic indicators (hyperlipidemia, bone density, wrinkling of the skin, thinning of the hair, muscle mass), inflammatory and oxidative stress biomarkers, telomere length, and synaptic function (Kirkpatrick et al., 2008; Nguyen et al., 2018). Large‐scale brain imaging studies also indicate faster structural brain aging in schizophrenia (Koutsouleris et al., 2014). Most recently, schizophrenia is reported to associate with different pace of epigenetic aging compared with healthy controls (Chrusciel et al., 2022; Wu et al., 2021).

Schizophrenia is known to have a strong genetic background: twin studies have shown its high heritability (80%) (Sullivan et al., 2003), and genome‐wide association studies (GWAS) have identified an extensive number of schizophrenia‐related SNPs (differences in a single DNA nucleotides) that explain 7–33% of the variance in liability to the disorder (Lee et al., 2012; Legge et al., 2021; Purcell et al., 2009). Interestingly, epigenetic aging and schizophrenia are found to have a partly shared genetic background, including genes related to the regulation of cell activation and development (Wu et al., 2021). Evidence on the association between polygenic risk for schizophrenia and epigenetic aging is limited and inconclusive. To the best of our knowledge, there are two studies on this very topic. In a multi‐cohort case–control study, female but not male schizophrenia patients with high polygenic risk for schizophrenia showed accelerated epigenetic aging in terms of differential phenotypic age (Levine clock), while no association was obtained with Horvath epigenetic aging (Ori et al., 2019). In another case–control study, the polygenic risk for schizophrenia was negatively correlated with DNAmAge gap but not with PhenoAge gap (Teeuw et al., 2021).

Neither of the studies, however, considered factors that might explain individual differences in the pace of epigenetic aging in psychosis‐susceptible populations. We hypothesized that, in individuals at risk for psychosis, social relationships may represent such a factor in protecting against accelerated aging. High‐risk cases who have good social relationships or who spend their time with familiar others are more likely to experience lesser stress, lower symptoms, recover symptomatically, or have a lower risk for conversion to psychosis (Addington et al., 2017; Cannon et al., 2008; Fett et al., 2022; Gee & Cannon, 2011; Ortega et al., 2019; Robustelli et al., 2017). Consistently, young people with early psychosis report that their social goals are to have larger networks, more peer relationships, and more social opportunities (Macdonald et al., 2005). A close and supportive social network, however, may not likely be successfully created and maintained by external attempts to “forced socialization” but, rather, by “patients' drive to establish new social contacts” (Giacco et al., 2016). Thus, a key factor appears to be an inner drive or readiness for social interactions, deriving from one's temperament‐ or personality‐based dispositions.

We examined, first, whether polygenic risk for schizophrenia (PRS_sch_) predicts the pace of epigenetic aging. Second, we examined whether personal dispositions toward active and emotionally close social interactions could protect against accelerated epigenetic aging in individuals with high PRS_sch_. We used data from the Young Finns Study, including a population‐based sample and a follow‐up of social dispositions from teenage years to middle age. The data provided possibilities to consider a wide array of potential confounders such as health behaviors, socioeconomic factors, and early family environment.

## 2 MATERIALS AND METHODS

### 2.1 Participants

The Young Finns Study (YFS) is an ongoing prospective follow‐up study that began in 1980 (baseline assessment), and follow‐ups have been conducted in 1983, 1986, 1989, 1992, 1997, 2001, 2007, 2012, and 2017. Altogether 4320 subjects were invited (born in 1962, 1965, 1968, 1971, 1974, or 1977), and 3596 of them participated in the baseline study. The sampling was designed to include a population‐based sample of noninstitutionalized Finnish children, representative with regard to most crucial sociodemographic factors. In practice, the sampling was conducted in collaboration of five Finnish universities with medical schools (i.e., Universities of Helsinki, Turku, Tampere, Oulu, and Kuopio). A more detailed description of the YFS can be found elsewhere (20).

The study design has been approved by the ethical committees of all the Finnish universities conducting the study. All the participants or their parents (participants aged <18 years) provided informed consent before participation. The Declaration of Helsinki has been followed throughout the study.

Of the 3596 participants, we first excluded 1885 participants who had no data on epigenetic clocks. Thereafter, in each analysis, we included participants who had data available on social dispositions in at least one measurement year and data available on covariates (sex, health behaviors, socioeconomic factors, early family environment). Accordingly, the sample size varied between 1088 and 1348 in the final analyses.

### 2.2 Measures

#### 2.2.1 Polygenic risk score for schizophrenia (PRS_sch_ )

Polygenic risk score for schizophrenia was calculated on the basis of the summary statistics of the most recent genome‐wide association study (GWAS) on schizophrenia that was conducted by Schizophrenia Working Group of the Psychiatric Genomics Consortium et al. and published in Nature (Consortium, 2014). Specifically, a weighted polygenic risk score (Igo Jr. et al., 2019) for every study subject was created by summing up each participant's schizophrenia‐associated risk alleles weighted by risk allele beta estimates (Consortium, 2014). Altogether 128 independent SNPs reaching genome‐wide significance in the schizophrenia GWAS were included in the PRS_sch_. More specifically, genotyping was done for 2556 samples using custom‐build Illumina Human 670 k BeadChip at Welcome Trust Sanger Institute. Sample call rate <0.95, excess heterozygosity, sex mismatch, cryptic relatedness (pi‐hat > 0.2), SNP call rate < 0.95, MAF < 0.01, and HWE *p*‐value < 1e^−6^ were used as quality control filters. After the quality control, there were 2443 samples and 546,677 genotyped SNPs available for further analysis. Genotype Imputation to 1000 Genomes reference was performed using SHAPEIT v1 for haplotype phasing and IMPUTE2 and 1000 Genomes March 2012 haplotypes for genotype imputation. SNPs with imputation information metric >0.3 were considered well‐imputed. This polygenic risk score for schizophrenia is shown to predict an increased likelihood of psychoses (Saarinen et al., 2022).

#### 2.2.2 Social dispositions

We assessed a broad repertoire of features and dispositions related to sociability and human relationships, including (1) sociability, (2) extraversion, (3) reward dependence, (4) cooperativeness, and (5) attachment security. Each disposition was assessed with a self‐report questionnaire widely used for research purposes.

*Sociability* assesses one's tendency to prefer and enjoy the presence of others over being alone. Sociability was measured in 1992, 1997, 2001, 2007, and 2012 (participants being 15–50 years) with the Sociability scale of the Emotionality, Activity, and Sociability Temperament Survey (EAS) (Buss & Plomin, 1975, 1986). The scale includes five items (e.g., “I like to be with people”) that are responded to with a 5‐point scale (1 = totally disagree, 5 = totally agree). The scale had adequate internal reliability (Cronbach's *α* = 0.77–0.87 in 1992–2012).

*Extraversion* includes dispositions toward warmth, gregariousness, assertiveness, activity, excitement seeking, and positive emotionality. Extraversion was measured in 2007 and 2012 with 12 items (“I really like to discuss with people”; Cronbach's *α* = 0.82–0.83 in 2007/2012) using Neuroticism‐Extraversion‐Openness Five‐Factor Inventory (NEO‐FFI; Costa & McCrae, 1992; McCrae & Costa Jr., 1988). Extraversion scores had high test–retest correlations between different measurement years (*r* = 0.79).

*Reward Dependence* assesses one's disposition to dependency on others' acceptance, sentimentality, and attachment to others. Reward Dependence was assessed with the Temperament and Character Inventory (TCI) (Cloninger et al., 1994). The scale of reward dependence (used in 1997, 2001, 2007, and 2012) includes 24 items responded with a 5‐point scale (1 = totally disagree, 5 = totally agree). The scale had high test–retest correlations between measurement years (*r* = 0.68–0.82) and high internal reliability (Cronbach's *α* = 0.79–0.80).

*Cooperativeness* assesses one's disposition toward social acceptance, empathy, helpfulness, compassion, and pure‐hearted conscience. Cooperativeness was also assessed with the TCI (Cloninger et al., 1994). The scale of Cooperativeness (used at the follow‐ups of 1997, 2001, and 2012) includes 42 items responded with a 5‐point scale (1 = totally disagree, 5 = totally agree). The scale was found to have good psychometric properties in terms of test–retest correlation (*r* = 0.64–0.73) and internal reliability (Cronbach's *α* = 0.90).

*Attachment security* refers to one's capacity to form and maintain emotionally close social relationships by trusting in others, seeking emotional support during distress, not constantly fearing others' rejection, and being comfortably alone if needed. Attachment security was measured in 2001, 2007, and 2012 using the Finnish version of the Relationship Questionnaire (Bartholomew & Horowitz, 1991). It consists of four statements, which are answered with a 7‐point Likert scale (1 = totally disagree, 7 = totally agree). The statements measure four attachment styles: secure, preoccupied, dismissing, and fearful (e.g., “I strive for relationships that are as close as possible, but others seem to avoid such closeness”). All items were scaled so that higher values referred to more secure attachment styles and summed together. Previous studies have reported high test–retest reliability during a 7‐year follow‐up for the Finnish version of the scale (Salo et al., 2011) and good predictive validity for the scale: attachment styles correlate with more mature personality development (Saarinen et al., 2018), depressive symptoms (Pesonen et al., 2004), and childhood maternal nurturance style (Salo et al., 2011).

For each social disposition, we calculated a mean score of the disposition over the follow‐up for all participants who had data available on the respective disposition in at least one measurement year. Pairwise correlations between different social dispositions ranged between *r* = 0.39–0.67, with strongest correlations being between extraversion and sociability (*r* = 0.67) and between extraversion and attachment (*r* = 0.57) and weakest correlations between cooperativeness and sociability (*r* = 0.39) and between cooperativeness and extraversion (*r* = 0.39).

#### 2.2.3 Indicators of epigenetic age acceleration

The samples for DNA methylation analysis were collected at the follow‐up point of 2011. Genome‐wide DNA methylation levels from whole blood were obtained with Illumina Infinium HumanMethylation450 BeadChip (*n* = 182) or Illumina Infinium MethylationEPIC BeadChip (*n* = 1529) following standard protocol by Illumina. Previously, it has been demonstrated that the lack of the clock‐CpGs on the EPIC array does not affect the utility of the epigenetic clock variables (McEwen et al., 2018). Preprocessing and normalization of the methylation data were conducted by the authors and have been described in detail elsewhere (Marttila et al., 2021).

Indicators of epigenetic age included in the study were the Horvath clock (Horvath, 2013), Hannum clock (Hannum et al., 2013), and their intrinsic and extrinsic derivatives, namely IEAA_Horvath_, IEAA_Hannum_ and EEAA_Hannum_ (Chen et al., 2016). Additionally, we included 2 s‐generation epigenetic clocks, that is, PhenoAge (Levine et al., 2018) and GrimAge (Lu et al., 2019). Generally, chronological age correlates strongly with epigenetic aging: for example, *r* = 0.94 with phenotypic age (Levine et al., 2018), *r* = 0.97 with Horvath DNA methylation age (Horvath, 2013), and *r* = 0.96 with Hannum DNA methylation age (Hannum et al., 2013). For the Horvath and Hannum clocks as well as PhenoAge and GrimAge, we utilized the measure of epigenetic age acceleration, which is defined as the residual that results from regressing epigenetic age on chronological age (Chen et al., 2016). These are denoted as AgeAccel_Horvath_, AgeAccel_Hannum_, AgeAccel_Pheno_, and AgeAccel_Grim_. Finally, we included a third‐generation measure for pace of aging, DunedinPACE (Belsky et al., 2022). All measures of epigenetic age or pace of aging were calculated by the authors according to the published methods described above. A histogram depicting the distribution of each variable of epigenetic aging in our data set can be found in Figure S1. Additionally, the pairwise correlations between the epigenetic clock variables are presented in Table S1.

Evidence suggests that the Hannum clock may be related to all‐cause mortality and aging‐related diseases more strongly than Horvath clocks (Fransquet et al., 2019), while the Horvath clock may have stronger associations with innate development such as puberty and menopause (Levine et al., 2018). A meta‐analysis suggested that the associations between Hannum/Horvath epigenetic clocks and risk of death are approximately similar between females and males and between different ethnic groups (Fransquet et al., 2019).

AgeAccel_Grim_ is known for its strong association with mortality (Föhr et al., 2021) and predicts severe somatic diseases and age‐related conditions (Lu et al., 2019; McCrory et al., 2021). AgeAccel_Pheno_ is found to relate to a wide scope of outcomes, including physical functioning, cognitive impairment, cancers, Alzheimer's disease, and all‐cause mortality (Levine et al., 2018; McCrory et al., 2021). Finally, DunedinPACE has high test–retest reliability (Belsky et al., 2022) and good predictive validity by predicting cognitive dysfunction, chronic conditions, Alzheimer's disease, and mortality in non‐clinical populations (Faul et al., 2023; Sugden et al., 2022).

#### 2.2.4 Covariates

Covariates included age, sex, participants' (2011) and their parents' (1980) socioeconomic factors (educational level, annual income), health behaviors (daily smoking status, BMI, alcohol consumption, physical activity), and qualities of early family environment (stressful life events and emotional family atmosphere in 1980). We included these factors as control variables in our analyses because of educational level and income (Hamlat et al., 2022; Simons et al., 2016), BMI, physical activity, alcohol consumption (Huang et al., 2019; Kresovich et al., 2021; Rosen et al., 2018), and psychosocial adversities in childhood family (Hamlat et al., 2021; Marini et al., 2020) are shown to correlate with epigenetic age acceleration. Also, there is evidence that psychosis risk correlates with lower socioeconomic status, less favorable health behaviors, and childhood adversities (Fusar‐Poli et al., 2017; Ruhrmann et al., 2010), implying their potential role as confounders. A more detailed description of the covariates is available in Data S1.

### 2.3 Statistical analyses

Data analysis was conducted using Stata SE 14.0. First, we used linear regression analyses to examine whether polygenic risk for schizophrenia (PRS_sch_) predicts indicators of epigenetic age acceleration. Separate models were estimated for each indicator: AgeAccel_Hannum_, EEAA_Hannum_, IEAA_Hannum_, IEAA_Horvath_, AgeAccel_Horvath_, AgeAccel_Pheno_, AgeAccel_Grim_, and DunedinPACE. Second, we examined whether social dispositions (extraversion, sociability, reward dependence, cooperativeness, and attachment security) moderate the associations of PRS_sch_ with indicators of epigenetic age acceleration. Each social disposition and its PRS_sch_‐interaction was added as a predictor separately.

Analyses were run with two different sets of covariates. Models 1 were adjusted for sex, array type (450 K or EPIC), and health behaviors (daily smoking status, body mass index [BMI], physical activity, and alcohol consumption). Models 2 were additionally adjusted for participants' and their parents' socioeconomic factors and early family environment (stressful life events and emotional atmosphere). To correct for multiple testing, we used false discovery rate (FDR) correction with Benjamini‐Hochberg procedure.

Finally, we examined attrition over the follow‐up by comparing included and dropped‐out participants with regard to study variables (using independent samples *t*‐tests and chi‐square tests).

## 3 RESULTS

Descriptive statistics of the sample are shown in Table 1. First, we examined attrition over the follow‐up: whether included and dropped‐out participants differed with regard to the study variables (for details, see Table S2). In summary, included participants had slightly higher scores in social dispositions: slightly higher extraversion, reward dependence, cooperativeness, and attachment security than dropped‐out participants. Also, included (vs. dropped‐out) participants had slightly more favorable health behaviors in terms of alcohol consumption and physical activity and also higher income. We did not find any attrition bias in PRS for schizophrenia or in most indicators of epigenetic age acceleration.

**TABLE 1:** Descriptive statistics of the sample.

	Mean ± *SD*	Frequency (%)	Measurement range
Age (2011)	42.0 ± 5.0		34–49
Sex (Female)		756 (56.1)	
Parents' educational level			
Comprehensive school		434 (32.7)	
Occupational school or high school		539 (40.6)	
Academic level		356 (26.8)	
Parents' annual income	5.0 ± 2.0		1–8
Educational level			
Comprehensive school		24 (1.8)	
Occupational school or high school		301 (22.6)	
Academic level		1007 (75.6)	
Annual income	7.5 ± 3.0		1–13
Daily smoking status		176 (13.1)	
Alcohol consumption	0.77 ± 1.1		0–10
Physical activity	9.1 ± 1.9		5–15
BMI	26.5 ± 4.8		17.5–58.5
Sociability^a^	3.5 ± 0.6		1–5
Extraversion^a^	3.4 ± 0.5		1–5
Reward Dependence^a^	3.3 ± 0.4		1–5
Cooperativeness^a^	3.8 ± 0.4		1–5
Attachment security^a^	5.3 ± 0.8		1–7
PRS for schizophrenia	0.0 ± 1.0		−3.4–3.2
AgeAccel_Hannum_	0.0 ± 4.2		−19.2–14.2
EEAA_Hannum_	0.0 ± 5.1		−18.7–16.8
IEAA_Hannum_	0.1 ± 3.7		−17.2–14.0
IEAA_Horvath_	0.2 ± 4.1		−19.6–18.9
AgeAccel_Horvath_	0.1 ± 4.2		−22.7–19.5
AgeAccel_Pheno_	0.2 ± 5.4		−17.5–20.1
AgeAccel_Grim_	−0.2 ± 3.6		−9.2–16.1
DunedinPACE	0.9 ± 0.1		0.61–1.3

*Note*: This table includes participants who were included in at least one analysis (*n* = 1348).

^a^ Mean over the follow‐up.

### 3.1 Main analyses

First, we examined the main effect of PRS on epigenetic age acceleration. Full results are presented in Table S3. Briefly, PRS_sch_ did not predict any indicator of epigenetic age acceleration in Models 1 (*p* = 0.240–0.961) or Models 2 (*p* = 0.216–0.824). Figure S2 presents the scatter plots between PRS and the variables of epigenetic aging. Also, there were no sex interactions of PRS_sch_ when predicting epigenetic age acceleration (*p* = 0.273–0.995).

Next, we examined whether social domains moderate the relationship between PRS_sch_ and epigenetic age acceleration. That is, we added an interaction effect between PRS_sch_ and each social disposition to the model. Table 2 presents the results of Models 1 (adjusted for sex, array type, daily smoking status, BMI, physical activity, and alcohol consumption). When predicting AgeAccel_Hannum_, EEAA_Hannum_, or IEAA_Hannum_, we found a significant interaction between PRS_sch_ and reward dependence (*B* = 0.76–1.17, *p* < 0.005 for all Hammun clocks), between PRS_sch_ and cooperation (*B* = 0.82, *p* = 0.011 for AgeAccel_Hannum_, *B* = 0.91, *p* = 0.020 for EEAA_Hannum_, *B* = 0.76, *p* = 0.009 for IEAA_Hannum_, respectively), between PRS_sch_ and extraversion (*B* = 0.55, *p* = 0.019, and *B* = 0.58, *p* = 0.041, and *B* = 0.49, *p* = 0.020, respectively), between PRS_sch_ and sociability (*B* = 0.51, *p* = 0.006, and *B* = 0.55, *p* = 0.016, and *B* = 0.49, *p* = 0.003, respectively), and between PRS_sch_ and attachment security (*B* = 0.37, *p* = 0.007, *B* = 0.41, *p* = 0.014, and *B* = 0.33, *p* = 0.008, respectively). All these associations remained statistically significant after applying FDR correction for multiple testing; the only exception was the interaction between PRS_sch_ and extraversion when predicting EEAA_Hannum_. Interactions between PRS_sch_ and social dispositions were mostly nonsignificant when predicting the other indicators of epigenetic age acceleration (IEAA_Horvath_, AgeAccel_Horvath_, AgeAccel_Pheno_, AgeAccel_Grim_, and DunedinPACE), regardless of applying or not applying FDR correction for multiple testing.

**TABLE 2:** Results of regression analyses when showing also the main effects of PRS and each social disposition when predicting indicators of epigenetic age acceleration.

	Social disposition in the model														
	Reward dependence (TCI) (*n* = 1269)			Cooperativeness (TCI) (*n* = 1232)			Extraversion (NEO‐FFI) (*n* = 1182)			Sociability (EAS) (*n* = 1316)			Attachment security (Bartholomew) (*n* = 1248)		
	*B*	*SE*	*p*	*B*	*SE*	*p*	*B*	*SE*	*p*	*B*	*SE*	*p*	*B*	*SE*	*p*
AgeAccel_Hannum_															
PRS	**−3.12**	**0.98**	0.015*	**−2.94**	**1.21**	0.015*	**−1.76**	**0.80**	**0.028**	**−1.62**	**0.65**	0.012*	**−1.83**	**0.73**	0.012*
Social predictor	**0.68**	**0.33**	**0.036**	0.32	0.32	0.320	0.13	0.23	0.571	0.20	0.19	0.280	0.15	0.14	0.293
Social predictor*PRS	**0.96**	**0.29**	0.001*	**0.82**	**0.32**	0.011*	**0.55**	**0.23**	0.019*	**0.51**	**0.19**	0.006*	**0.37**	**0.14**	0.007*
EEAA_Hannum_															
PRS	**−3.79**	**1.19**	0.002*	**−3.28**	**1.48**	**0.027**	−1.88	0.98	0.055	**−1.75**	**0.80**	**0.028**	**−2.07**	**0.90**	**0.021**
Social predictor	0.78	0.40	0.050	0.62	0.40	0.115	0.27	0.28	0.331	0.32	0.23	0.173	0.27	0.17	0.119
Social predictor*PRS	**1.17**	**0.36**	0.001*	**0.91**	**0.39**	0.020*	**0.58**	**0.28**	**0.041**	**0.55**	**0.23**	0.016*	**0.41**	**0.17**	0.014*
IEAA_Hannum_															
PRS	**−2.48**	**0.89**	0.005*	**−2.76**	**1.09**	0.012*	**−1.59**	**0.73**	**0.029**	**−1.60**	**0.59**	0.006*	**−1.68**	**0.66**	0.012*
Social predictor	0.47	0.30	0.113	0.00	0.29	0.992	0.00	0.21	0.990	0.03	0.17	0.855	0.05	0.13	0.672
Social predictor*PRS	**0.76**	**0.26**	0.004*	**0.76**	**0.29**	0.009*	**0.49**	**0.21**	0.020*	**0.49**	**0.17**	0.003*	**0.33**	**0.12**	0.008*
IEAA_Horvath_															
PRS	−1.92	0.99	0.053	−1.60	1.24	0.195	−0.92	0.81	0.253	−0.32	0.65	0.623	−1.25	0.74	0.093
Social predictor	0.10	0.33	0.751	−0.53	0.33	0.111	0.23	0.23	0.323	0.15	0.19	0.441	0.01	0.14	0.969
Social predictor*PRS	0.56	0.29	0.057	0.42	0.33	0.203	0.25	0.23	0.280	0.09	0.19	0.638	0.23	0.14	0.102
AgeAccel_Horvath_															
PRS	**−2.30**	**1.00**	**0.021**	−1.43	1.25	0.251	−0.97	0.81	0.233	−0.45	0.66	0.500	−1.38	0.75	0.066
Social predictor	0.07	0.33	0.823	−0.38	0.33	0.257	0.27	0.23	0.242	0.17	0.19	0.385	0.07	0.14	0.639
Social predictor*PRS	**0.68**	**0.30**	**0.022**	0.37	0.33	0.257	0.27	0.24	0.249	0.13	0.19	0.501	0.25	0.14	0.069
AgeAccel_Pheno_															
PRS	−2.18	1.28	0.088	**−3.94**	**1.58**	0.013*	−0.70	1.04	0.499	−0.78	0.84	0.353	−1.57	0.96	0.102
Social predictor	0.12	0.43	0.772	−0.51	0.42	0.222	0.04	0.30	0.895	0.24	0.25	0.336	0.19	0.18	0.307
Social predictor*PRS	0.65	0.38	0.089	**1.04**	**0.42**	0.013*	0.20	0.30	0.509	0.21	0.24	0.373	0.29	0.18	0.105
AgeAccel_Grim_															
PRS	−0.13	0.65	0.847	−0.60	0.81	0.455	0.63	0.53	0.236	0.60	0.43	0.162	0.05	0.48	0.913
Social predictor	**0.47**	**0.22**	**0.033**	**0.47**	**0.22**	**0.030**	0.21	0.15	0.161	**0.28**	**0.13**	**0.026**	0.01	0.09	0.905
Social predictor*PRS	0.01	0.19	0.961	0.13	0.21	0.529	−0.21	0.15	0.173	−0.20	0.12	0.109	−0.03	0.09	0.757
DunedinPACE															
PRS	0.01	0.02	0.688	−0.01	0.03	0.836	0.02	0.02	0.226	0.02	0.01	0.109	0.01	0.02	0.472
Social predictor	0.00	0.01	0.561	0.01	0.01	0.237	0.00	0.00	0.942	0.00	0.00	0.318	0.00	0.00	0.736
Social predictor*PRS	0.00	0.01	0.755	0.00	0.01	0.775	−0.01	0.00	0.255	−0.01	0.00	0.120	0.00	0.00	0.515

*Note*: Statistically significant (*p* < 0.05) associations are bolded. An asterisk (*) indicates statistical significance after FDR correction for multiple testing. Models were adjusted for sex, array type, and health behaviors (daily smoking status, BMI, physical activity, and alcohol consumption).

**FIGURE 1:**
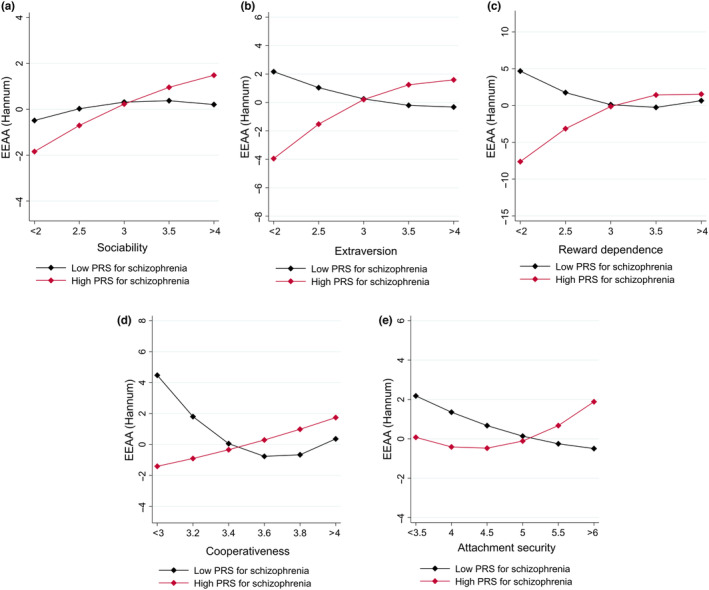
Model‐predicted values of EEAA_Hannum_ at different levels of (a) sociability, (b) extraversion, (c) reward dependence, (d) cooperativeness, and (e) attachment security. Those values were plotted separately for subjects with low PRS (PRS being at least one SD below the mean in our sample) or high PRS (PRS being at least one SD above the mean in our sample). Adjusted for sex, array type, and health behaviors (smoking status, BMI, alcohol consumption, physical activity). For illustrative purposes, we allowed PRS_sch_ to have curvilinear interactions with the social predictors.

The findings are illustrated in Figure 1a–e, where we plotted model‐predicted values of EEAA_Hannum_ at different levels of social dispositions, separately for participants with low PRS (PRS at least one SD below the sample mean) and high PRS (PRS at least one SD above the sample mean). Briefly, participants with high PRS_sch_ had lower epigenetic age acceleration at lower (vs. higher) levels of extraversion, sociability, attachment security, reward dependence, and cooperativeness. On the contrary, participants with low PRS_sch_ appeared to have lower epigenetic age acceleration at higher (vs. lower) levels of extraversion, attachment security, reward dependence, and cooperativeness.

The findings were replicated in Models 2 that were further adjusted for participants' and their parents' socioeconomic factors and early emotional family environment (stressful life events and emotional atmosphere; see Table S4). Also, almost all the results remained after FDR correction for multiple testing (the only exceptions were the interactions between PRS_sch_ and extraversion when predicting EEAA_Hannum_ or IEAA_Hannum_).

### 3.2 Sensitivity analyses

First, we reran the interaction analyses so that participants with nonaffective psychotic disorders were excluded from the sample (collection of psychiatric diagnoses is described in Data S1). Again, all the main results were replicated. That is, we found an interaction effect between PRS_sch_ and each social disposition when predicting AgeAccel_Hannum_ (*B* = 0.40–0.90, *p* = 0.002–0.023), EEAA_Hannum_ (*B* = 0.43–1.11, *p* = 0.002–0.048), or IEAA_Hannum_ (*B* = 0.37–0.70, *p* = 0.005–0.022).

Second, as a minority of the data set was analyzed with a 450K array, we reran the interaction analyses so that only EPIC array data were included. The findings were mostly replicated, also after applying FDR correction for multiple testing (see Table S5). The only exceptions were that the interaction between PRS_sch_ and reward dependence became significant when predicting AgeAccelPheno (*B* = 1.03, *p* = 0.010) and the interaction between PRS_sch_ and extraversion became non‐significant when predicting AgeAccel_Hannum_ (*p* = 0.063) or EEAA_Hannum_ (*p* = 0.097).

## 4 DISCUSSION

PRS_sch_ did not have any main effect on any indicator of epigenetic aging. We found, however, that social dispositions modified the associations of PRS_sch_ with AgeAccel_Hannum_, EEAA_Hannum_, or IEAA_Hannum_, indicating that individuals with high PRS_sch_ seemed to have higher pace of epigenetic aging at higher (vs. lower) levels of extraversion, sociability, attachment security, reward dependence, and cooperativeness. Thus, the interaction was replicated across all of our social indicators. A rather opposite pattern was obtained in participants with low PRS_sch_. When predicting the other indicators of epigenetic aging (IEAA_Horvath_, AgeAccel_Horvath_, AgeAccel_Pheno_, AgeAccel_Grim_, and DunedinPACE), none of the associations was significant.

We found that social dispositions modified the associations of PRS_sch_ with epigenetic aging. The findings indicate that individuals with high PRS_sch_ may have an *accelerated* pace of epigenetic aging if they also have a strong personal need to have social company and spend time with others, to be dependent on others' acceptance, to behave in cooperation with others, or to have an emotionally close bonding to others. Although they are in contradiction with our hypotheses, the findings are in line with some previous studies. It has been found that patients with psychotic disorders may be less likely to report feelings of loneliness despite lesser social contacts (Giacco et al., 2016) and less likely to perceive a lack of friendships as a problem (Harley et al., 2012). Also, secure attachment is found not to protect against depressive or anxiety symptoms in individuals at risk for psychosis (Russo et al., 2018). Thus, it seems that social contacts may not necessarily support health and well‐being in individuals at risk for psychosis.

Against our hypotheses, the results imply that frequent social interactions may be strenuous for individuals at risk for psychosis. A potential explanation may lie in their weaker capacity to cope with social interactions. Previous studies have found that individuals at clinical risk for psychosis may have a stronger bias to perceive hostility in others' behavior (An et al., 2010), a lower awareness of social inferences (Glenthøj et al., 2016), slight impairments in their theory of mind (Piskulic et al., 2016; Thompson et al., 2011), and a higher disposition to paranoid interpretations after being socially excluded (Lincoln et al., 2018). Accordingly, there is evidence that a perceived lack of control in social situations may provoke distress in youth at clinical risk for psychosis (Millman et al., 2017).

A second explanation for our results may be that some individuals at risk for psychosis may have a deviant composition in their social network, due to accumulation of social risk factors within individuals. There is evidence that individuals with psychosis spectrum are more likely to live in more socially fragmented neighborhoods (Solmi et al., 2020), to perceive bullying victimization in their social networks (Braun et al., 2022), to report experiences of discrimination or stigmatization (Colizzi et al., 2020), or initiate substance use with their acquaintances (Archie et al., 2013). In addition, individuals at psychosis spectrum are more likely to report having less diverse social networks (Robustelli et al., 2017): for example, they may perceive fellow users of mental health services as their friends (Harley et al., 2012) or report healthcare professionals as members of their social network (Pernice‐Duca, 2008). Thus, individuals at risk for psychosis may, in some cases, live in a social network where their interpersonal relationships are not fully emotionally supportive.

Our interaction analyses identified associations with Hannum clocks but not with Horvath clocks or DunedinPACE. Also, two previous studies have reported associations of PRS_sch_ with some epigenetic clocks but not with others. In the first study, PRS_sch_ was negatively correlated with the DNAmAge gap but not with the PhenoAge gap (Teeuw et al., 2021), and, in the second study, female patients with high PRS_sch_ displayed accelerated aging in differential phenotypic age (Levine clock) but not in Horvath age (Ori et al., 2019). Since evidence is still very limited, more research is needed to more deeply understand why PRS_sch_ may correlate with only certain epigenetic clocks. Overall, Hannum clocks may be related to all‐cause mortality and aging‐related diseases more strongly than Horvath clocks (Fransquet et al., 2019), whereas the Horvath clock may have stronger associations with innate maturation such as menopause or puberty (Levine et al., 2018). While schizophrenia is related to shortened lifetime expectancy and premature mortality (Plana‐Ripoll et al., 2019, 2020), the significant associations with Hannum clocks seem plausible. Moreover, the genes regulated by epigenetic clocks include also schizophrenia‐linked genes, related to cell activation and development (Wu et al., 2021), and Hannum clock is found to capture more cell‐extrinsic aging with moderate correlation with cell compositions (Hannum et al., 2013).

While we found significant interactions between PRS_sch_ and social dispositions, we did not find any significant main effect of PRS_sch_ on epigenetic aging. Overall, our study in combination with previous evidence (Chrusciel et al., 2022; Wu et al., 2021) implies that different pace of epigenetic aging may be more evident in schizophrenia patients vs. in individuals at genetic risk for the disorder (without the disorder). The onset of schizophrenia may have effects on epigenetic aging via multiple mechanisms. Specifically, the onset of the disorder commonly results in sick leaves, antipsychotic medications, unemployment periods, and narrowed social network that, in turn, seems to correlate with pace of epigenetic aging on the basis of preliminary evidence (Beach et al., 2022; Das, 2022; Du et al., 2022; Freni‐Sterrantino et al., 2022; Li et al., 2023).

Our attrition analyses showed that there was not any drop‐out bias in PRS for schizophrenia or in most indicators of epigenetic age acceleration. Thus, our data collection captured quite well subpopulations with different levels of epigenetic aging or genetic risk factors for schizophrenia. Included participants had, however, slightly higher scores in social dispositions: slightly higher extraversion, reward dependence, cooperativeness, and attachment security than dropped‐out participants. Hence, our results cannot be directly generalized to populations with very low social dispositions.

When calculating the PRS_sch_, we used the genome‐wide association study (GWAS) on schizophrenia that was most recent at that time, conducted by Schizophrenia Working Group of the Psychiatric Genomics Consortium et al. and including a total of 128 schizophrenia‐related SNPs (Consortium, 2014). Since then, a more recent GWAS study on schizophrenia has been published (Trubetskoy et al., 2022) and, also, novel statistical methods have been developed to enhance calculations (Privé et al., 2021). Nevertheless, recent reviews have emphasized that “PRS will never be able to establish or definitively predict a diagnosis of common complex conditions” such as schizophrenia (Murray et al., 2021) and that “even with the rapid expansion of the psychiatric genetic knowledge base, pure genetic prediction in clinical psychiatry appears to be out of reach in the near future” (Fusar‐Poli et al., 2022). Thus, a recently recommended way of improving predictive accuracy is to combine PRSs with other risk factors (Fusar‐Poli et al., 2022; Murray et al., 2021). Consistently, the focus of our study was to consider a broad array of psychosocial factors along with PRS.

Previously, a number of social functioning interventions have been developed for individuals with psychosis spectrum (Devoe et al., 2019). Also, there have been recommendations to increase those individuals' “drive to establish new social contacts” (Giacco et al., 2016). Individuals at genetic risk for psychosis, however, have on average lower temperament‐based drive for social contacts (Saarinen et al., in press) that, in turn, appears to correlate with a decelerated pace of epigenetic aging. Thus, as noted previously, social withdrawal may act as a protective strategy against excessive arousal in psychosis spectrum individuals who perceive social contacts overwhelming (Palumbo et al., 2015). Hence, although individuals at risk for psychosis have a limited number of social contacts (Gayer‐Anderson & Morgan, 2013), it may not indicate a distressing discrepancy between their ideal and actualized social activities but may rather reflect their lower temperament‐based social drive. Additionally, instead of aiming to increase an intrinsic social drive, it is important to focus on providing concrete stress regulation strategies on how to cope with possible feelings of distress or uncontrollability in social situations. Finally, individuals at risk for psychosis may need support to form relationships outside health care settings and outside potential delinquent networks so that social contacts, when taking place, could be reciprocal and emotionally safe.

## AUTHOR CONTRIBUTIONS

M.K., O.R., J.H., L.K.‐J., and T.L. contributed to data collection. S.M., P.P.M., L.‐P.L., N.M., E.R., and E.S. contributed to data preprocessing. A.S. conducted the statistical analyses and wrote an initial draft. All authors contributed to commenting and writing of the manuscript.

## CONFLICT OF INTEREST STATEMENT

The authors declare no competing financial interests in relation to the work described.

## Supporting information


Data S1:

## Data Availability

The Cardiovascular Risk in Young Finns (YFS) dataset comprises health‐related participant data, and their use is therefore restricted under the regulations on professional secrecy (Act on the Openness of Government Activities, 612/1999) and on sensitive personal data (Personal Data Act, 523/1999, implementing the EU data protection directive 95/46/EC). Due to these legal restrictions, the data from this study cannot be stored in public repositories or otherwise made publicly available. However, data access may be permitted on a case by case basis upon request. Data sharing outside the group is done in collaboration with YFS group and requires a data‐sharing agreement. Investigators can submit an expression of interest to the chairman of the publication committee (Prof. Mika Kähönen, Tampere University, Finland, mika.kahonen@tuni.fi).
