# Supplementary material for: Polygenic risk for schizophrenia, social dispositions, and pace of epigenetic aging: Results from the Young Finns Study
Source: Aging Cell. 2023 Nov 29;23(3):e14052. doi: 10.1111/acel.14052 (PMC10928579; doi:10.1111/acel.14052)
Supplement: Supplementary file 1 — Data S1: [file ACEL-23-e14052-s001.docx]

**Supplementary Material**

**S1. Measurement of socioeconomic factors**

Socioeconomic factors included participants’ (2011) and their parents’ (1980) annual income and educational level. Educational level was classified into three categories (1 = comprehensive school, i.e., the nine first school years; 2 = high school or occupational school; 3 = academic level). In case mother’s and father’s educational level differed from each other, we used the higher educational level. Annual income in childhood was assessed with a 8-point scale (1 = less than 15 000 Finnish mark; 8 = more than 100 000 Finnish mark) and in adulthood with a 11-point scale (1 = less than 5 000 €; 11 = more than 60 000 €).

**S2. Assessment of health behaviors**

**Smoking** was assessed in 2011 with self-report questionnaire in 2011 (1 = daily smoking; 6 = having never smoked). We formed a dichotomous variable (0 = not daily smoking; 1 = daily smoking).

**Alcohol consumption** was assessed in 2011 by asking the participants to report their consumption of 1/3 l cans or bottles of beer, glasses (12 cl) of wine, and 4 cl shots of liquor or strong alcohol during the last week. These amounts are comparable to approximately 14 g of alcohol. We calculated a sum score of the items of different beverages consumed within the past week. A similar index of alcohol consumption has been used also previously (e.g., Juonala et al., 2009).

**Physical activity** was assessed in 2011 with five items: (i) “How much breathlessness and sweating do you experience when you engage in sport or physical activity?” (1=not at all; 3=a lot); (ii) “How often do you engage in sport or physical activity so that you get out of breath and sweat?” (1=never; 6=daily); (iii) “How many hours per week do you usually engage in sport or physical activity so that you get out of breath and sweat?” (1=not at all; 6=7 hours or more); (iv) “How much time do you usually spend in one session of sport of physical activity?” (1=less than 20 minutes; 4=more than 60 minutes); (v) “Do you participate in organized physical activity (e.g. in sport club)?” (1=not at all; 4=several hours per week). A total score of the items was calculated for each measurement year. This index of physical activity has been used previously (e.g., Tammelin et al., 2014).

**S3. Assessment of childhood family environment**

**The cumulative score of emotional family atmosphere** in 1980/1983 included the following factors: emotional distance between the child and parent, parental intolerance toward the child, strict discipline toward the child, parental life dissatisfaction, mother’s or father’s mental disorder (no/yes), and mother’s or father’s frequent alcohol intoxication (ranging from “1=never” to “8=daily”). This score has been used also previously (24).

*Emotional distance between the parent and child* was evaluated with a four-item questionnaire (e.g., “The child is emotionally important for me”, ”I can realize myself with the child”). The items were responded with a 5-point scale (e.g., 1 = little, 5 = much). *Parental intolerance toward the child* was evaluated with a three-item scale (“I get nervous when spending time with the child”, “The child is a burden in challenging situations”, “The child consumes my time too much”). The items were responded with a 5-point scale (1 = frequently, 5 = never). *Strict discipline toward the child* was measured with a three-item scale (“Disciplinary actions are often needed at home due to child’s aggressiveness”; “Disciplinary actions do not affect the child enough”; “Disciplinary actions are necessary in the rearing of the child”). The items measuring parenting have been used also previously (66, 67).

*Parental life satisfaction* was assessed with a three-item questionnaire measuring parent’s satisfaction in three life sectors: as a parent, spouse, and employee. The items were responded with a 5-point scale (1 = satisfied, 5 = dissatisfied). This questionnaire has been adapted from the Operation Family Study questionnaire (68) and has been used also previously (69, 70).

**The cumulative score of stressful life events** in 1980/1983 included the following factors: change of residence, number of change of school, parental divorce (whether parents living together or separated), mother’s or father’s death, mother’s or father’s hospitalization within the past 12 months (number of days in hospital, ranging from “1 = no days” to “5 = more than 30 days”), and child’s hospitalization due to sickness or accident (no/yes). This cumulative score has been used also previously (24).

**S4. Assessment of psychiatric diagnoses**

Participants’ psychiatric diagnoses over their lifespan were collected up to 2017 (when participants were 40–55 years old) from the Care Register for Health Care (<https://thl.fi/en/web/thlfi-en/statistics-and-data/data-and-services/register-descriptions/care-register-for-health-care>). The register includes disorders that have required hospital care. There is evidence that ca. 97 % of psychotic disorders are covered by the register (71). Diagnoses were given in accordance with the diagnostic classification at that time (ICD-8, ICD-9, or ICD-10). Conversion of ICD-diagnoses to DSM-IV diagnoses has been described elsewhere (72). Non-affective psychoses included the diagnoses of DSM-IV 295 and 297–298. The register has been used also in previous research (73).

**Supplementary Table 1.** Pairwise correlations between the epigenetic clock variables and chronological age in our sample (*n* = 1348).

|  | **1.** | **2.** | **3.** | **4.** | **5.** | **6.** | **7.** | **8.** | **9.** |
| --- | --- | --- | --- | --- | --- | --- | --- | --- | --- |
| **1. AgeAccel_Hannum_** | **-** |  |  |  |  |  |  |  |  |
| **2. EEAA_Hannum_** | 0.97 | - |  |  |  |  |  |  |  |
| **3. IEAA_Hannum_** | 0.87 | 0.76 | - |  |  |  |  |  |  |
| **4. IEAA_Horvath_** | 0.30 | 0.26 | 0.37 | - |  |  |  |  |  |
| **5. AgeAccel_Horvath_** | 0.35 | 0.33 | 0.34 | 0.95 | - |  |  |  |  |
| **6. AgeAccel_Pheno_** | 0.43 | 0.48 | 0.25 | 0.29 | 0.33 | - |  |  |  |
| **7. AgeAccel_Grim_** | 0.11 | 0.16 | -0.01 | -0.01 | 0.01 | 0.31 | - |  |  |
| **8. DunedinPACE** | 0.12 | 0.18 | -0.01 | -0.03 | -0.02 | 0.36 | 0.48 | **-** |  |

**Supplementary Table 2.** Differences between included and dropped-out participants in the main study variables. Note: n.s. = non-significant

|  | Mean difference  (included *vs*. dropped-out) | Test statistic | *p* |
| --- | --- | --- | --- |
| Age (2011) | 42.0 vs. 41.1 | *t* = 5.40 | < 0.001 |
| Sex (Female) | 56.1 % *vs*. 47.9 % | χ² = 27.77 | < 0.001 |
| Parents’ educational level |  |  | n.s. |
| Adulthood educational level |  |  | n.s |
| Parents’ annual income | 4.96 vs. 4.70 | *t* = 3.90 | < 0.001 |
| Adulthood annual income | 7.47 vs. 7.16 | *t* = 2.10 | < 0.05 |
| Daily smoking status | 13.1 % vs. 20.0 % | χ² = 16.18 | < 0.001 |
| Alcohol consumption | 0.8 vs. 0.9 | *t* = 2.38 | < 0.05 |
| Physical activity | 9.1 vs. 8.8 | *t* = 3.33 | < 0.001 |
| BMI |  |  | n.s. |
| Sociability^1^ |  |  | n.s. |
| Extraversion^1^ | 3.41 vs. 3.35 | *t* = 2.67 | < 0.01 |
| Reward Dependence^1^ | 3.33 vs. 3.28 | *t* = 3.38 | < 0.001 |
| Cooperativeness^1^ | 3.76 vs. 3.70 | *t* = 4.25 | < 0.001 |
| Attachment security^1^ | 5.31 vs. 5.17 | *t* = 4.08 | < 0.001 |
| PRS for schizophrenia |  |  | n.s. |
| AgeAccel_Hannum_ |  |  | n.s. |
| EEAA_Hannum_ |  |  | n.s. |
| IEAA_Hannum_ |  |  | n.s. |
| IEAA_Horvath_ |  |  | n.s. |
| AgeAccel_Horvath_ |  |  | n.s. |
| AgeAccel_Pheno_ |  |  | n.s. |
| AgeAccel_Grim_ | -0.17 vs. 0.68 | *t* = 3.87 | < 0.001 |
| DunedinPACE | 0.94 vs. 0.96 | *t* = 3.03 | < 0.01 |
|  | | | |

**Supplementary Table 3.** Results of regression analyses. Estimates (B) with standard errors (SE) of the PRS when predicting indicators of epigenetic age acceleration.

|  | Model 1 (n = 1348) | | |  | Model 2 (n = 1227) | | |
| --- | --- | --- | --- | --- | --- | --- | --- |
|  | B | *SE* | *p* |  | B | *SE* | *p* |
| AgeAccel_Hannum_ | 0.12 | 0.11 | 0.276 |  | 0.14 | 0.12 | 0.216 |
| EEAA_Hannum_ | 0.12 | 0.13 | 0.387 |  | 0.15 | 0.14 | 0.309 |
| IEAA_Hannum_ | 0.10 | 0.10 | 0.337 |  | 0.12 | 0.11 | 0.273 |
| IEAA_Horvath_ | -0.02 | 0.11 | 0.821 |  | -0.04 | 0.12 | 0.720 |
| AgeAccel_Horvath_ | -0.02 | 0.11 | 0.851 |  | -0.04 | 0.12 | 0.705 |
| AgeAccel_Pheno_ | -0.06 | 0.14 | 0.676 |  | -0.09 | 0.15 | 0.562 |
| AgeAccel_Grim_ | -0.09 | 0.07 | 0.240 |  | -0.09 | 0.08 | 0.248 |
| DunedinPACE | 0.00 | 0.00 | 0.667 |  | 0.00 | 0.00 | 0.948 |
| Models 1 were adjusted for sex, array type, and health behaviors (daily smoking status, BMI, physical activity, alcohol consumption).  Models 2 were additionally for participants’ and their parents’ socioeconomic factors and early emotional family environment (stressful life events and emotional atmosphere). | | | | | | | |

**Supplementary Table 4.** Results of full-adjusted regression analyses. Estimates (B) with standard errors (SE) of the interaction effect between PRS and each social disposition, when predicting indicators of epigenetic age acceleration. Statistically significant (p < 0.05) associations are bolded. An asterisk (*) indicates statistical significance after FDR correction for multiple testing.

|  | Social predictor in the model | | | | | | | | | | | | | | | | | | | | |
| --- | --- | --- | --- | --- | --- | --- | --- | --- | --- | --- | --- | --- | --- | --- | --- | --- | --- | --- | --- | --- | --- |
|  | Reward Dependence  (TCI) (n = 1159) | | |  | Cooperativeness  (TCI) (n = 1124) | | |  | Extraversion  (NEO-FFI) (n = 1088) | | |  |  | Sociability  (EAS) (n = 1200) | | |  | Attachment security  (Bartholomew) (n = 1142) | | | |
|  | B | *SE* | *p* |  | B | *SE* | *p* |  | B | *SE* | *p* |  |  | B | *SE* | *p* |  | B | *SE* | *p* | |
| AgeAccel_Hannum_ |  |  |  |  |  |  |  |  |  |  |  |  |  |  |  |  |  |  |  |  | |
| PRS | **-3.47** | **1.03** | **0.001*** |  | **-3.54** | **0.19** | **0.006*** |  | **-1.84** | **0.85** | **0.030** |  |  | **-1.57** | **0.69** | **0.023** |  | **-1.93** | **0.78** | **0.013*** | |
| Social disposition | 0.56 | 0.34 | 0.103 |  | 0.21 | 0.35 | 0.552 |  | 0.07 | 0.25 | 0.782 |  |  | 0.23 | 0.20 | 0.266 |  | 0.08 | 0.15 | 0.585 | |
| Social disposition*PRS | **1.09** | **0.31** | **< 0.001*** |  | **0.99** | **0.34** | **0.004*** |  | **0.58** | **0.25** | **0.018*** |  |  | **0.50** | **0.20** | **0.011*** |  | **0.39** | **0.14** | **0.007*** | |
| EEAA_Hannum_ |  |  |  |  |  |  |  |  |  |  |  |  |  |  |  |  |  |  |  |  | |
| PRS | **-4.16** | **1.26** | **0.001*** |  | **-4.08** | **1.58** | **0.010*** |  | **-2.07** | **1.04** | **0.046** |  |  | **-1.71** | **0.85** | **0.045** |  | **-2.30** | **0.95** | **0.016*** | |
| Social disposition | 0.63 | 0.42 | 0.137 |  | 0.45 | 0.42 | 0.288 |  | 0.15 | 0.30 | 0.630 |  |  | 0.33 | 0.25 | 0.184 |  | 0.18 | 0.19 | 0.325 | |
| Social disposition*PRS | **1.30** | **0.37** | **0.001*** |  | **0.14** | **0.42** | **0.007*** |  | **0.65** | **0.30** | **0.030** |  |  | **0.55** | **0.24** | **0.025** |  | **0.47** | **0.18** | **0.009*** | |
| IEAA_Hannum_ |  |  |  |  |  |  |  |  |  |  |  |  |  |  |  |  |  |  |  |  | |
| PRS | **-2.84** | **0.93** | **0.002*** |  | **-3.09** | **1.17** | **0.008*** |  | **-1.59** | **0.77** | **0.039** |  |  | **-1.44** | **0.63** | **0.022** |  | **-1.61** | **0.71** | **0.023** | |
| Social disposition | 0.34 | 0.31 | 0.277 |  | -0.10 | 0.31 | 0.762 |  | -0.01 | 0.23 | 0.955 |  |  | 0.06 | 0.18 | 0.751 |  | 0.00 | 0.14 | 0.972 | |
| Social disposition*PRS | **0.88** | **0.29** | **0.002*** |  | **0.85** | **0.31** | **0.006*** |  | **0.50** | **0.22** | **0.027** |  |  | **0.45** | **0.18** | **0.012*** |  | **0.32** | **0.13** | **0.014*** | |
| IEAA_Horvath_ |  |  |  |  |  |  |  |  |  |  |  |  |  |  |  |  |  |  |  |  | |
| PRS | **-2.44** | **1.04** | **0.019*** |  | -2.41 | 1.31 | 0.067 |  | -1.46 | 0.85 | 0.088 |  |  | -0.70 | 0.69 | 0.313 |  | **-1.55** | **0.79** | **0.049** | |
| Social disposition | 0.09 | 0.35 | 0.803 |  | **-0.72** | **0.35** | **0.042** |  | 0.12 | 0.25 | 0.631 |  |  | 0.11 | 0.20 | 0.588 |  | -0.02 | 0.15 | 0.895 | |
| Social disposition*PRS | **0.72** | **0.31** | **0.020** |  | 0.63 | 0.35 | 0.071 |  | 0.41 | 0.25 | 0.097 |  |  | 0.19 | 0.20 | 0.326 |  | 0.28 | 0.15 | 0.053 | |
| AgeAccel_Horvath_ |  |  |  |  |  |  |  |  |  |  |  |  |  |  |  |  |  |  |  |  | |
| PRS | **-2.70** | **1.04** | **0.010*** |  | -2.20 | 1.32 | 0.095 |  | -1.45 | 0.86 | 0.093 |  |  | -0.76 | 0.70 | 0.276 |  | **-1.59** | **0.79** | **0.045** | |
| Social disposition | 0.05 | 0.35 | 0.876 |  | -0.68 | 0.35 | 0.057 |  | 0.15 | 0.25 | 0.548 |  |  | 0.12 | 0.21 | 0.558 |  | 0.03 | 0.15 | 0.853 | |
| Social disposition*PRS | **0.80** | **0.31** | **0.010*** |  | 0.57 | 0.35 | 0.101 |  | 0.41 | 0.25 | 0.101 |  |  | 0.21 | 0.20 | 0.285 |  | **0.29** | **0.15** | **0.048** | |
| AgeAccel_Pheno_ |  |  |  |  |  |  |  |  |  |  |  |  |  |  |  |  |  |  |  |  | |
| PRS | -2.36 | 1.34 | 0.078 |  | **-4.64** | **1.67** | **0.006*** |  | -0.87 | 1.10 | 0.428 |  |  | -1.44 | 0.89 | 0.105 |  | -1.91 | 1.01 | 0.060 | |
| Social disposition | -0.03 | 0.45 | 0.944 |  | -0.71 | 0.45 | 0.116 |  | -0.14 | 0.32 | 0.662 |  |  | 0.10 | 0.26 | 0.699 |  | 0.14 | 0.20 | 0.476 | |
| Social disposition*PRS | 0.69 | 0.40 | 0.082 |  | **1.22** | **0.44** | **0.006*** |  | 0.25 | 0.32 | 0.431 |  |  | 0.40 | 0.25 | 0.120 |  | 0.35 | 0.19 | 0.066 | |
| AgeAccel_Grim_ |  |  |  |  |  |  |  |  |  |  |  |  |  |  |  |  |  |  |  |  | |
| PRS | 0.33 | 0.66 | 0.623 |  | -0.08 | 0.83 | 0.927 |  | 0.88 | 0.55 | 0.109 |  |  | 0.67 | 0.44 | 0.128 |  | 0.13 | 0.50 | 0.798 | |
| Social disposition | **0.49** | **0.22** | **0.029** |  | **0.65** | **0.22** | **0.004*** |  | 0.30 | 0.16 | 0.061 |  |  | **0.31** | **0.13** | **0.018*** |  | -0.04 | 0.09 | 0.669 | |
| Social disposition*PRS | -0.12 | 0.20 | 0.530 |  | 0.00 | 0.22 | 0.989 |  | -0.28 | 0.16 | 0.080 |  |  | -0.22 | 0.13 | 0.088 |  | -0.04 | 0.09 | 0.669 | |
| DunedinPACE |  |  |  |  |  |  |  |  |  |  |  |  |  |  |  |  |  |  |  |  | |
| PRS | 0.01 | 0.02 | 0.597 |  | -0.01 | 0.03 | 0.812 |  | 0.19 | 0.01 | 0.295 |  |  | 0.02 | 0.01 | 0.128 |  | 0.01 | 0.02 | 0.569 | |
| Social disposition | 0.00 | 0.01 | 0.740 |  | 0.01 | 0.01 | 0.054 |  | 0.00 | 0.01 | 0.762 |  |  | 0.01 | 0.00 | 0.134 |  | 0.00 | 0.00 | 0.738 | |
| Social disposition*PRS | 0.00 | 0.01 | 0.634 |  | 0.00 | 0.01 | 0.775 |  | -0.01 | 0.10 | 0.312 |  |  | -0.01 | 0.00 | 0.124 |  | 0.00 | 0.00 | 0.598 | |
| Models were adjusted for sex, array type, health behaviors (daily smoking status, BMI, physical activity, alcohol consumption), participants’ and their parents’ socioeconomic factors, and early emotional family environment (stressful life events and emotional atmosphere).  *Note:* Main effects of PRS_sch_ and each social domain were also included in the models but excluded from this table due to limited space. | | | | | | | | | | | | | | | | | | | | |  |

**Supplementary Table 5.** Results of interaction analyses when predicting indicators of epigenetic age so that only EPIC array data were included. Statistically significant (p < 0.05) interactions are bolded. Associations with changed statistical significance (when compared to the main analyses) are marked with green color. An asterisk (*) indicates statistical significance after FDR correction for multiple testing.

|  | Social disposition in the model | | | | | | | | | | | | | | | | | | | |
| --- | --- | --- | --- | --- | --- | --- | --- | --- | --- | --- | --- | --- | --- | --- | --- | --- | --- | --- | --- | --- |
|  | Reward Dependence (TCI) (n = 1108) | | |  | Cooperativeness  (TCI) (n = 1073) | | |  | Extraversion  (NEO-FFI) (n = 953) | | |  |  | Sociability  (EAS) (n = 1149) | | |  | Attachment security  (Bartholomew) (n = 1087) | | |
|  | B | *SE* | *p* |  | B | *SE* | *p* |  | B | *SE* | *p* |  |  | B | *SE* | *p* |  | B | *SE* | *p* |
| AgeAccel_Hannum_ |  |  |  |  |  |  |  |  |  |  |  |  |  |  |  |  |  |  |  |  |
| PRS | **-3.02** | **1.06** | **0.004*** |  | **-3.11** | **1.32** | **0.018*** |  | -1.48 | 0.86 | 0.086 |  |  | **-1.50** | **0.69** | **0.030** |  | **-1.84** | **0.79** | **0.020*** |
| Social disposition | 0.61 | 0.35 | 0.084 |  | 0.26 | 0.35 | 0.453 |  | 0.07 | 0.25 | 0.771 |  |  | 0.13 | 0.21 | 0.539 |  | 0.13 | 0.15 | 0.387 |
| Social disposition*PRS | **0.94** | **0.31** | **0.003*** |  | **0.87** | **0.35** | **0.013*** |  | 0.47 | 0.25 | 0.063 |  |  | **0.48** | **0.20** | **0.015*** |  | **0.37** | **0.15** | **0.012*** |
| EEAA_Hannum_ |  |  |  |  |  |  |  |  |  |  |  |  |  |  |  |  |  |  |  |  |
| PRS | **-3.79** | **1.29** | **0.003*** |  | **-3.78** | **1.61** | **0.019*** |  | -1.61 | 1.05 | 0.125 |  |  | -1.62 | 0.85 | 0.057 |  | **-2.01** | **0.96** | **0.037** |
| Social disposition | 0.71 | 0.43 | 0.094 |  | 0.59 | 0.42 | 0.166 |  | 0.26 | 0.30 | 0.455 |  |  | 0.27 | 0.25 | 0.288 |  | 0.27 | 0.18 | 0.150 |
| Social disposition*PRS | **1.18** | **0.38** | **0.002*** |  | **1.06** | **0.43** | **0.013*** |  | 0.51 | 0.31 | 0.097 |  |  | **0.52** | **0.24** | **0.032** |  | **0.41** | **0.18** | **0.024** |
| IEAA_Hannum_ |  |  |  |  |  |  |  |  |  |  |  |  |  |  |  |  |  |  |  |  |
| PRS | **-2.48** | **0.95** | **0.009*** |  | **-2.90** | **1.18** | **0.014*** |  | -1.50 | 0.77 | 0.052 |  |  | **-1.78** | **0.62** | **0.004*** |  | **-1.85** | **0.71** | **0.009*** |
| Social disposition | 0.43 | 0.31 | 0.169 |  | -0.02 | 0.31 | 0.957 |  | -0.05 | 0.22 | 0.813 |  |  | -0.06 | 0.18 | 0.738 |  | 0.01 | 0.14 | 0.931 |
| Social disposition*PRS | **0.76** | **0.28** | **0.007*** |  | **0.79** | **0.31** | **0.012*** |  | **0.46** | **0.22** | **0.042** |  |  | **0.54** | **0.18** | **0.003*** |  | **0.36** | **0.13** | **0.007*** |
| IEAA_Horvath_ |  |  |  |  |  |  |  |  |  |  |  |  |  |  |  |  |  |  |  |  |
| PRS | **-2.22** | **1.05** | **0.035** |  | -2.01 | 1.32 | 0.128 |  | -1.21 | 0.85 | 0.154 |  |  | -0.63 | 0.68 | 0.351 |  | -1.46 | 0.78 | 0.062 |
| Social disposition | 0.10 | 0.35 | 0.768 |  | -0.40 | 0.35 | 0.252 |  | 0.19 | 0.24 | 0.447 |  |  | 0.04 | 0.20 | 0.828 |  | -0.07 | 0.15 | 0.649 |
| Social disposition*PRS | **0.64** | **0.31** | **0.041** |  | 0.51 | 0.35 | 0.143 |  | 0.32 | 0.25 | 0.195 |  |  | 0.16 | 0.19 | 0.399 |  | 0.26 | 0.15 | 0.079 |
| AgeAccel_Horvath_ |  |  |  |  |  |  |  |  |  |  |  |  |  |  |  |  |  |  |  |  |
| PRS | **-2.41** | **1.08** | **0.025** |  | -1.86 | 1.36 | 0.171 |  | -1.11 | 0.87 | 0.202 |  |  | -0.57 | 0.70 | 0.419 |  | -1.41 | 0.80 | 0.079 |
| Social disposition | 0.04 | 0.36 | 0.908 |  | -0.28 | 0.36 | 0.438 |  | 0.22 | 0.25 | 0.374 |  |  | 0.08 | 0.21 | 0.686 |  | 0.02 | 0.15 | 0.896 |
| Social disposition*PRS | **0.70** | **0.32** | **0.028** |  | 0.48 | 0.36 | 0.182 |  | 0.30 | 0.25 | 0.238 |  |  | 0.15 | 0.20 | 0.442 |  | 0.25 | 0.15 | 0.091 |
| AgeAccel_Pheno_ |  |  |  |  |  |  |  |  |  |  |  |  |  |  |  |  |  |  |  |  |
| PRS | **-3.38** | **1.35** | **0.013*** |  | **-5.17** | **1.68** | **0.002*** |  | -0.45 | 1.09 | 0.679 |  |  | -0.67 | 0.88 | 0.445 |  | -1.54 | 1.01 | 0.126 |
| Social disposition | -0.03 | 0.45 | 0.948 |  | -0.48 | 0.44 | 0.280 |  | 0.14 | 0.32 | 0.663 |  |  | 0.31 | 0.26 | 0.232 |  | 0.22 | 0.19 | 0.247 |
| Social disposition*PRS | **1.03** | **0.40** | **0.010*** |  | **1.395** | **0.46** | **0.002*** |  | 0.15 | 0.32 | 0.647 |  |  | 0.21 | 0.25 | 0.405 |  | 0.30 | 0.19 | 0.108 |
| AgeAccel_Grim_ |  |  |  |  |  |  |  |  |  |  |  |  |  |  |  |  |  |  |  |  |
| PRS | -0.38 | 0.71 | 0.590 |  | -0.84 | 0.88 | 0.340 |  | 0.54 | 0.57 | 0.346 |  |  | 0.46 | 0.46 | 0.321 |  | 0.04 | 0.52 | 0.946 |
| Social disposition | **0.53** | **0.23** | **0.025** |  | **0.52** | **0.23** | **0.027** |  | 0.23 | 0.16 | 0.158 |  |  | **0.30** | **0.14** | **0.026** |  | 0.01 | 0.10 | 0.896 |
| Social disposition*PRS | 0.09 | 0.21 | 0.674 |  | 0.20 | 0.23 | 0.398 |  | -0.18 | 0.17 | 0.266 |  |  | -0.15 | 0.13 | 0.242 |  | -0.02 | 0.10 | 0.805 |
| DunedinPACE |  |  |  |  |  |  |  |  |  |  |  |  |  |  |  |  |  |  |  |  |
| PRS | 0.00 | 0.02 | 0.992 |  | -0.02 | 0.03 | 0.588 |  | 0.02 | 0.02 | 0.358 |  |  | 0.02 | 0.01 | 0.201 |  | 0.02 | 0.02 | 0.351 |
| Social disposition | -0.01 | 0.01 | 0.464 |  | 0.01 | 0.01 | 0.236 |  | 0.00 | 0.01 | 0.756 |  |  | 0.01 | 0.00 | 0.220 |  | 0.00 | 0.00 | 0.991 |
| Social disposition*PRS | 0.00 | 0.01 | 0.916 |  | 0.01 | 0.01 | 0.499 |  | 0.00 | 0.01 | 0.405 |  |  | 0.00 | 0.00 | 0.238 |  | 0.00 | 0.00 | 0.402 |
| Models were adjusted for sex, array type, and health behaviors (daily smoking status, BMI, physical activity, alcohol consumption).  *Note:* Main effects of PRS and each social domain were also included in the models but excluded from this table due to limited space. | | | | | | | | | | | | | | | | | | | | |

**Supplementary Figure 1.** Distribution of each indicator of epigenetic ageing (histogram).

**Supplementary Figure 2.** A scatter plot of polygenic risk score for schizophrenia and each variable of epigenetic ageing.
